# Supplementary material for: Higher hypnotic suggestibility is associated with the lower EEG signal variability in theta, alpha, and beta frequency bands
Source: PLoS One. 2020 Apr 9;15(4):e0230853. doi: 10.1371/journal.pone.0230853 (PMC7145105; doi:10.1371/journal.pone.0230853)
Supplement: S1 Data — (ZIP) [file pone.0230853.s001.zip › DE_Features/readMe.pdf]

Supplementary Materials: DE features

Higher Hypnotic Suggestibility Is Associated with the Lower EEG Signal Variability in Theta, Alpha, and Beta Frequency Bands

Soheil Keshmiri[1*], Maryam Alimardani[1,2], Masahiro Shiomi[1], Hidenobu Sumioka[1], Hiroshi Ishiguro[1,3], Kazuo Hiraki[4]

1 Advanced Telecommunications Research Institute International (ATR), Kyoto, Japan 2 Department of Cognitive Science and Artificial Intelligence, Tilburg University, the Netherlands 3 Graduate School of Engineering Science, Osaka University, Japan

4 Department of General Systems Studies, Tokyo University, Japan

* soheil@atr.jp

DE features

The folder "DE_Features" contains the DE features that were used in this study. It includes 14 subfolders that correspond to EEG channels through which the brain activity of HIGH and LOW suggestible participants was recorded.

Each subfolder, in turn, includes 10 .mat files, oner per participant. Each .mat file comprises a Matlab structure "values" with two fields:

1) values.X: This is a 10 X 3 matrix, per participant, which contains the DE features of theta, alpha, and beta frequency bands (in the same order) of an individual. 10 refers to the number of hypnotic suggestions, per participant.

2) values.label: This is 10 X 1 row vector with all whose entries either 1 (if the participant belonged to HIGH group) or -1 (if the participant belonged to LOW group).
